# Effectiveness of Teeth Whitening after Regenerative Endodontics Procedures: An In Vitro Study

**DOI:** 10.3390/jcm11237016

**Published:** 2022-11-28

**Authors:** Irini Fagogeni, Joanna Metlerska, Tomasz Falgowski, Maciej Górski, Mariusz Lipski, Alicja Nowicka

**Affiliations:** 1 Doctoral Studies of the Faculty of Dentistry, Pomeranian Medical University, 70-204 Szczecin, Poland; 2 Department of Conservative Dentistry and Endodontics, Pomeranian Medical University, 70-204 Szczecin, Poland; 3 General, Minimally Invasive and Gastrointestinal Surgery Department, Pomeranian Medical University, 70-204 Szczecin, Poland; 4 University Dental Clinic, Pomeranian Medical University, 70-204 Szczecin, Poland; 5 Department of Preclinical Conservative Dentistry and Preclinical Endodontics, Pomeranian Medical University, 70-204 Szczecin, Poland

**Keywords:** regenerative endodontic treatment, bleaching, discolouration, whitening, carbamide peroxide, platelet-rich fibrin

## Abstract

Discolouration resulting from regenerative endodontic procedures may have a negative impact on the quality of life of treated patients; therefore, it is recommended to minimize this risk by selecting appropriate scaffolds and barrier materials, and if discolouration occurs, the use of a simple, cost-effective and minimally invasive technique, such as whitening, should be considered. This in vitro study aimed to evaluate tooth discolouration after two- and single-visit regenerative endodontic procedures and the effectiveness of subsequent whitening procedures with carbamide peroxide. Two hundred bovine incisors were included in this study and divided into twenty groups based on the tested material combinations. Two groups were control groups, one with saline and the other group with blood. In the 12 groups, the experiment was designed to be consistent with the two-visit regenerative endodontic procedures. Triple antibiotic paste or calcium hydroxide were placed in the root canal, and then scaffolds (e.g., blood or platelet-rich fibrin) and barrier materials (Biodentine, OrthoMTA or MTA Repair HP) were applied after rinsing the disinfectant pastes. In the six groups that corresponded to the single-visit regenerative endodontic procedure, the use of a disinfectant paste was omitted. Subsequently, the specimens were bleached twice with carbamide peroxide at a 7-day interval. Colour change measurements were performed using a spectrophotometer (VITA Easyshade Compact 5.0, VITA Zahnfabrik, Bad Säckingen, Germany). Statistical analysis was performed with the Kruskal–Wallis H test, the independent *t*-test and *t*-test for related samples. Tooth discolouration was noticed after two- and single-visit regenerative endodontic procedures, except for the platelet-rich fibrin+MTA Repair HP group. After the first and second whitening procedures, all of the tested two- and single-visit regenerative endodontic procedures groups showed a change in the colour of the crown, which was noticeable to the naked eye (∆E > 3.3). When analysing the ∆E value between the first and second bleaching procedures, no changes in the colour of teeth were visually noticed in the calcium hydroxide and platelet-rich fibrin +MTA Repair HP groups (∆E < 3.3). Single-visit regenerative endodontic procedures are suggested if possible; however, if two-visit regenerative endodontic procedures are performed, it is recommended to use calcium hydroxide as the disinfectant paste because of the lower staining potential. In the context of discolouration, platelet-rich fibrin is advisable for use as a scaffold. The whitening procedure is worth considering, but does not guarantee a return to the original tooth colour, especially when triple antibiotic paste is used.

## 1. Introduction

Regenerative endodontic treatment is a dynamically developing field in endodontics that allows regeneration of damaged dentin and root structures, as well as the pulp–dentin complex [1] in immature permanent teeth with pulpal necrosis (with or without apical periodontitis) [2]. Continued root development and maturation are important advantages of regenerative endodontic procedures [3]; however, tooth discolouration is an unfavourable outcome of these procedures [4,5,6]. According to the American Association of Endodontists, regenerative endodontic procedures involve the removal of the infected pulp, disinfection of the root canal with disinfectant pastes, promotion of bleeding into the canal system by over-instrumenting, and placement of the barrier material over a blood clot [7]. If intracanal bleeding is not attainable, platelet-rich fibrin can be used as a scaffold [8,9].

Many authors have noticed crown discolouration after regenerative endodontic procedures, which may negatively affect the quality of life of treated patients [10]; therefore, it is recommended to reduce this risk as much as possible. To minimise the risk of discolouration, the American Association of Endodontists suggests sealing the pulp chamber with a dentin bonding agent prior to the application of the disinfectant pastes, although some authors have shown that this technique counteracts, but does not eliminate, the problem [11,12].

The American Association of Endodontists recommends the use of calcium hydroxide as a disinfectant paste. Calcium hydroxide may limit discolouration after regenerative endodontic procedures [4]; however, Chen et al. [13] reported that it is not indifferent for tooth colour. Triple antibiotic paste should be placed below the cementoenamel junction [7]. The concentration of triple antibiotic paste should be reduced, or minocycline, which has the greatest staining potential, should be replaced with another antibiotic (e.g., clindamycin, amoxicillin and cefaclor) or omitted [7]; nevertheless, in vitro [14,15,16] and in vivo [11] studies have shown that despite following the American Association of Endodontists recommendations, discolouration could not always be avoided.

It has been noted that white ProRoot MTA (Dentsply, Tulsa, OK, USA) has a strong staining potential [17], and it is recommended to use Biodentine (Septodont, Saint-Maur-des-Fossés, France) or EndoSequence BC RRM-Fast Set Putty (Brasseler, Savannah, GA, USA) as barrier materials, especially if regenerative endodontic procedures are performed in the anterior teeth [7]. If tooth discolouration occurs despite the use of the aforementioned techniques, the use of a simple, cost-effective, minimally invasive technique, such as whitening, should be considered. Teeth whitening is a process that lightens the colour of the tooth [18]. Materials that affect the colour of the tooth are most often organic compounds with conjugated chains of alternating single or double bonds, often containing heteroatoms and phenyl and carbonyl rings, and are referred to as chromophores [19,20]. In the context of chemical processes, bleaching occurs due to the breakdown of double bonds, cleavage of conjugated chains or oxidation of other chemical molecules in the conjugated chain [19]. This is due to the action of hydrogen peroxide, which can be used directly as a bleaching agent or can be released from sodium perborate or carbamide peroxide [21]. Hydrogen peroxide diffuses into the tooth structure, dissociates and generates oxygen free radicals [20]. As a result of the reaction of highly reactive oxygen radicals with chromophores, products are formed that are polar and have a lower molecular weight and are easily removed from the tooth in an aqueous environment [22]. Additionally, de novo products are brighter [20].

Teeth whitening after regenerative endodontic procedures can be a challenge for clinicians, especially with regard to the selection of the material used and its concentration, as well as the method of performing the procedure. In a group of patients younger than 18 years of age, the 2012 Cosmetic Products Safety Amendment Regulations allowed the use of <0.1% hydrogen peroxide and other compounds or mixtures that release hydrogen peroxide [23,24]. Yet, a new regulation in the General Dental Council’s Position Statement on Tooth Whitening asserts that products that contain or release between 0.1% and 6% hydrogen peroxide cannot be used in patients younger than 18 years of age, unless the purpose of bleaching is to treat or prevent disease [25]. There are no designated guidelines on how bleaching procedures should be performed [26]. Researchers have bleached discoloured teeth using different techniques, including internal [27,28,29] and external [30] techniques, or both [30]. The internal bleaching technique is similar to the walking bleach technique, which is used to bleach non-vital teeth. The bleaching material is placed in the pulp chamber, and the newly created pulp-like tissue in the root canal is protected by a barrier material [16]. Different types of bleaching agents have been used in previous studies, including hydrogen peroxide [31], carbamide peroxide [30,32] and sodium perborate [11,27,28], or a mixture of these materials [33,34]. Sodium perborate is prohibited in Europe [35] and is classified as toxic to reproduction, carcinogenic, or mutagenic.

Several case reports have shown that whitening of discoloured teeth after regenerative endodontic procedures is achievable and satisfactory for the patient [36,37], and even the colour of adjacent or other teeth can be achieved [27], although this is not a fully predictable procedure. There is a lack of extensive in vitro studies on large research materials that analyse the whitening of discoloured teeth after single- and two-visit regenerative endodontic procedures in the context of different disinfectant pastes, scaffolds, and barrier materials. Several authors have analysed bleached teeth after single- or two-visit regenerative endodontic procedures in which blood and a selected barrier material are placed into the root canal [31,32,34], but no studies have evaluated bleaching after regenerative endodontic procedures during which platelet-rich fibrin is used. Additionally, most of the authors used a high concentration of whitening agents that did not meet the requirements for dental treatment in patients younger than 18 years of age. All these factors were considered in the present study, which was performed using extensive researched materials and was designed to evaluate bleaching after the use of various disinfectant pastes and subsequent scaffolds (blood and platelet-rich fibrin) and barrier materials, including the latest calcium silicate-based cements, e.g., MTA Repair HP. This study aimed to examine tooth crown discolouration after two- and single-visit regenerative endodontic procedures and the effectiveness of the whitening procedure. The null hypotheses assume that, first, tooth tissue discolouration occurs after regenerative endodontic procedures, and second, whitening would be achievable.

## 2. Materials and Methods

### 2.1. Ethics Statements

**Figure 1:**
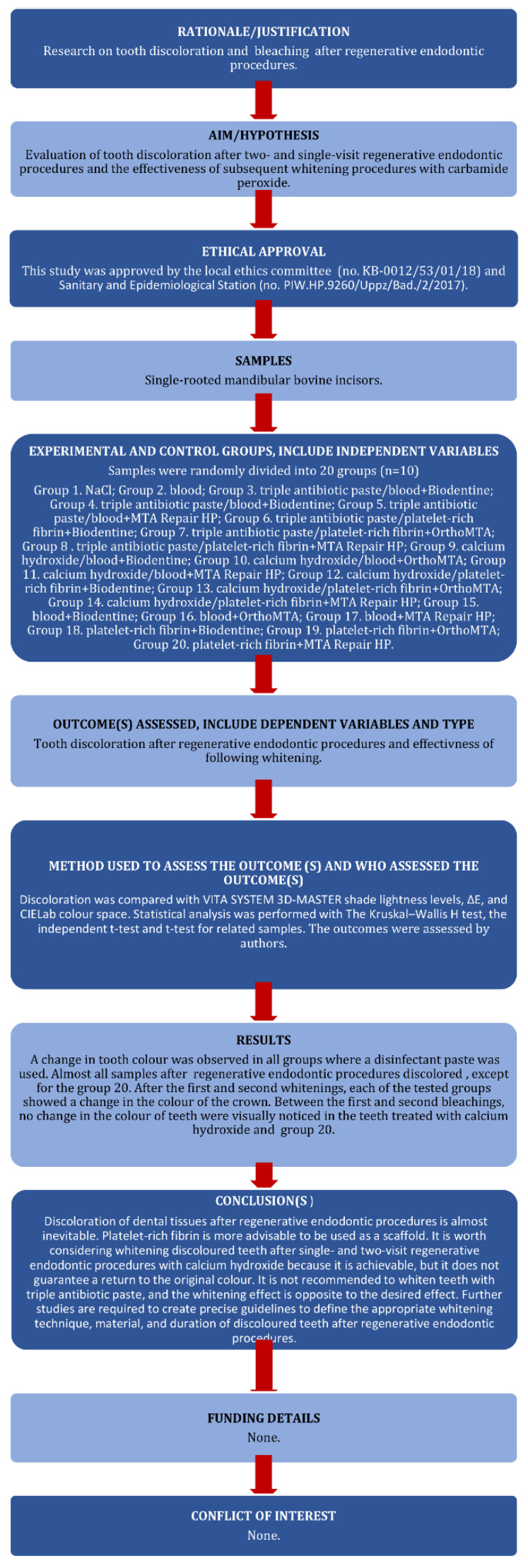
The PRILE 2021 flowchart.

The manuscript of this laboratory study has been written according to the Preferred Reporting Items for Laboratory Studies in Endodontology (PRILE) 2021 guidelines [38]. The PRILE 2021 flowchart is presented in Figure 1. The use of single-rooted mandibular bovine incisors in this study was approved by the local ethics committee (no. KB-0012/53/01/18). The teeth were collected from bovine heads from a local meat processing plant that were to be disposed of. The procedure was approved by the local Sanitary and Epidemiological Station (No. PIW.HP.9260/Uppz/Bad./2/2017).

### 2.2. Sample Preparation

**Figure 2:**
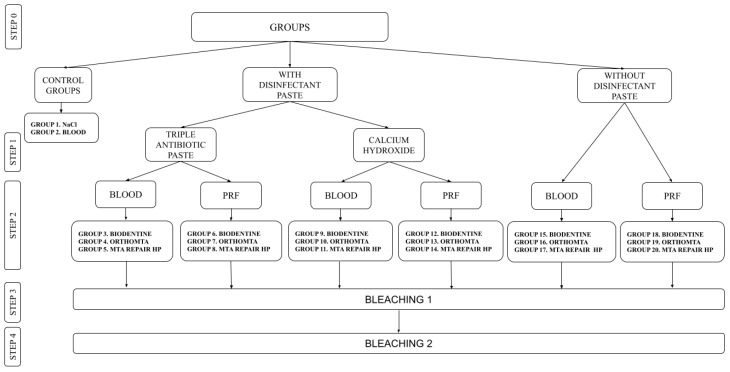
Description of the experimental groups. Abbreviations: PRF, platelet-rich fibrin.

After the extraction of 230 teeth, the specimens were stored in 1% chloramine solution for 24 h at 22 °C. All teeth were cleaned of plaque, sediment and stains using an ultrasonic scaler, and polished with water and pumice paste. Thirty teeth were excluded because of dental abnormalities or caries. Two-hundred teeth were included in the study and prepared for further research, similar to Shokouhinejad et al.’s study [39]. Endodontic access was prepared, and the pulp was removed from the pulp chambers and root canals using a barbed broach. To simulate immature teeth and standardise the length of the roots, the apical part of the root was removed 15 mm from the cementoenamel junction with a diamond bur in a high-speed hand piece under water cooling, and 4 mm of the apical part was sealed with glass ionomer cement (Kromoglass 2, LASCOD, Sesto Fiorentino, Italy). The root canals were shaped using 1#–6# Gates Glidden drills. The canals were irrigated with 1.5% NaOCl (20 mL/5 min), followed by 17% ethylenediaminetetraacetic acid (EDTA) (20 mL/5 min) and dried with absorbent paper points. The teeth were randomly divided into 20 groups (*n* = 10) (Figure 2). Two of the groups were control groups in which the root canals were filled with 0.9% NaCl (group 1) or blood (group 2). The internal walls of the pulp chambers of the remaining specimens were sealed with a dentin bonding agent (Tokuyama Universal Bond, Tokuyama Dental, Tokyo, Japan), according to the manufacturer’s instructions.

Twelve study groups were designed to undergo two-visit regenerative endodontic procedures. In these groups, disinfectant pastes were applied to the root canal below the cementoenamel junction using a syringe. Six groups were treated with triple antibiotic paste, and the other six groups were treated with calcium hydroxide. Triple antibiotic paste consisted of ciprofloxacin, metronidazole, and minocycline in equal proportions (1:1:1) and was prepared with 0.9% NaCl to obtain a 5 mg/mL paste. Calcium hydroxide was prepared according to the manufacturer’s instructions (Biopulp, Chema Elektromet, Rzeszów, Poland). After 3 weeks, disinfectant pastes were rinsed out from the canal with 17% EDTA (20 mL/5 min) and dried with absorbent paper points. Next, blood or platelet-rich fibrin was applied to the canal 4 mm below the cementoenamel junction, and 3 mm of calcium silicate-based cement was placed on the scaffold. The barrier materials used were Biodentine, MTA Repair HP, and OrthoMTA, and their compositions and preparations are described in Table 1. Finally, after rinsing the triple antibiotic paste from the canal, three groups were treated with blood and the following barrier material: Biodentine (group 3), OrthoMTA (group 4), or MTA Repair HP (group 5), and the next three groups were treated with platelet-rich fibrin and the following barrier material: Biodentine (group 6), OrthoMTA (group 7), or MTA Repair HP (group 8). The same procedure was performed for the samples with calcium hydroxide. After disinfection, calcium hydroxide was replaced with blood and the following barrier material: Biodentine (group 9), OrthoMTA (group 10), or MTA Repair HP (group 11) in the three groups, and in the next three groups, teeth were treated with platelet-rich fibrin and the following barrier material: Biodentine (group 12), OrthoMTA (group 13), or MTA Repair HP (group 14).

In six groups, disinfectant pastes were not used, which is consistent with the single-visit regenerative endodontic procedures. Scaffold and intracanal barrier materials were applied to the root canal, in a similar way to the following previous groups: blood + Biodentine (group 15), blood + OrthoMTA (group 16), blood + MTA Repair HP (group 17), platelet-rich fibrin + Biodentine (group 18), platelet-rich fibrin + OrthoMTA (group 19), and platelet-rich fibrin + MTA Repair HP (group 20). Blood was collected from a healthy author. Cement was placed on the scaffold 15 min after the blood clot had formed. For platelet-rich fibrin preparation, human blood was collected from the cephalic vein in sterile tubes and centrifuged for 14 min at 1500 rpm using a Dr. Choukroun DUO Quattro PRF centrifuge (Mount Pleasant, SC, USA). After centrifugation, three layers formed inside the tube, including platelet-poor plasma, platelet-rich fibrin, and erythrocytes from which the platelet-rich fibrin layer was selected. After application of the scaffolds and barrier materials, the cavity was sealed with glass ionomer cement. After the setting of the cement was confirmed, the access cavity was filled with glass ionomer cement for 4 weeks. After 4 weeks, glass ionomer cement and 2 mm of the barrier material were removed, and cervical sealing with glass ionomer cement was performed. Then, carbamide peroxide (Peroxidon, Chema—Elektromet, Rzeszów, Poland) was placed into the pulp chamber and covered with a small cotton pellet, and the access cavity was closed with glass ionomer cement. The bleaching agent was applied for 1 week. The whitening procedure was performed twice at a 7-day interval. During the study, the samples were stored in an incubator (220 V, 50 Hz; Carbolab Electronic, Warsaw, Poland) at 100% humidity and 37 °C.

### 2.3. Colour Assessment

To measure the colour of the specimens, two blinded operators used a spectrophotometer (VITA Easyshade Compact 5.0, VITA Zahnfabrik, Bad Säckingen, Germany). The specimens were placed at the level of the measuring tip of the device. Then, impression materials molds were made (Aquasil Soft Putty, Dentsply DeTrey, Konstanz, Germany) on the labial surface of the teeth, with an oval hole cut-out in the area of the cervical third of each tooth with a diameter of 6 mm, corresponding to the diameter of the spectrophotometer’s measuring probe tip. Before the measurement, the specimens were dried for 3 s with a blower. Each measurement was repeated thrice, and the spectrophotometer was calibrated before each measurement. The measurement time points for each step are listed in Table 2.

The use of a spectrophotometer allowed the measurement of the values of L, a and b and evaluation of the shade of the tooth in accordance with the international VITA SYSTEM 3D-MASTER shade systems. As suggested by the manufacturer (Vita-Zahnfabrik, Bad Säckingen, Germany), the 26 shades of the Toothguide 3D Master were grouped into 5 groups of lightness levels [40] and are presented in Table 3. The authors performed qualitative measurements and assigned them to the appropriate lightness levels.

To evaluate the exact colour changes, the authors carried out quantitative measurements of L, a, and b and used the ΔE values in the CIELab colour space. ΔE is the difference between the following and initial values, and was calculated according to the formula

$$\Delta E=\sqrt{{\left(\Delta L\right)}^{2}+{\left(\Delta a\right)}^{2}+{\left(\Delta b\right)}^{2}}$$

L is the lightness, which ranges from 0 (black) to 100 (white), a represents red (+) to green (−), and b represents yellow (+) to blue (−).

A calculated ΔE value of ≥3.3 is considered as clinically perceptible [41]. Photographs of samples were taken at each stage of the study with a specially designed EyeSpecial C-II device (Shofu, Inc., Kyoto, Japan), which is a professional camera created for dental practice with autoflash adjustment to reflect true colour. Photographs were taken in a specially designed form that allows the same distance between the camera and the sample to be obtained. Photos were taken in the automatic mode provided by the manufacturer. No further modifications or enhancements were applied to the images.

### 2.4. Statistical Analysis

Statistical analyses were performed using NumPy [42], SciPy [43], Pandas [44], and Scikit-learn [45] libraries for the Python programming language. Graphs were generated using the Matplotlib [46] and Seaborn [47] Python libraries. Basic statistical descriptive tests were performed, and data are presented as the mean and standard deviation. The Kruskal–Wallis H test was used to evaluate the qualitative results from the VITA SYSTEM 3D-MASTER shade lightness levels. The independent *t*-test and *t*-test for related samples were used to compare the numerical values between the groups and steps in the quantitative analysis. Hierarchical clustering was performed using the Ward method with Euclidean affinity. A statistically significant test result threshold was set at *p* < 0.005 [48].

## 3. Results

**Figure 3:**
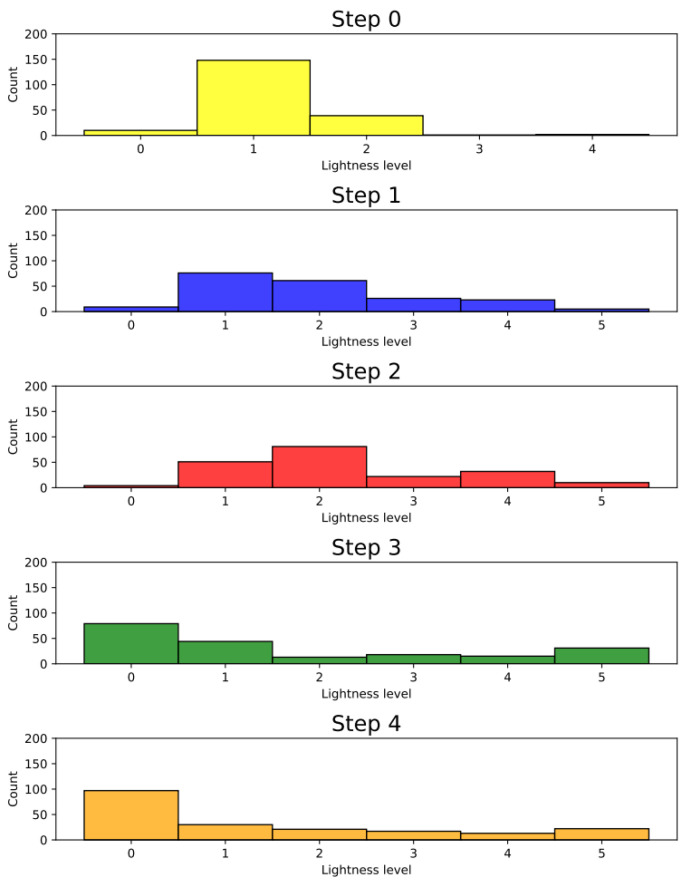
Lightness level distribution of the VITA SYSTEM 3D-MASTER for each step.

**Figure 4:**
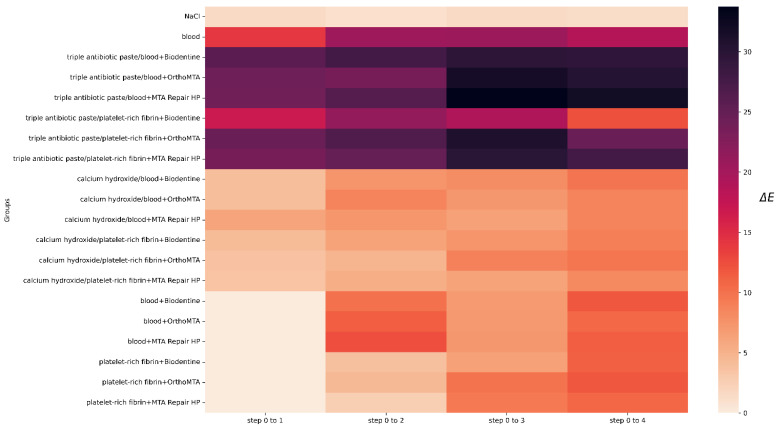
Values of ∆E for all groups and steps.

**Figure 5:**
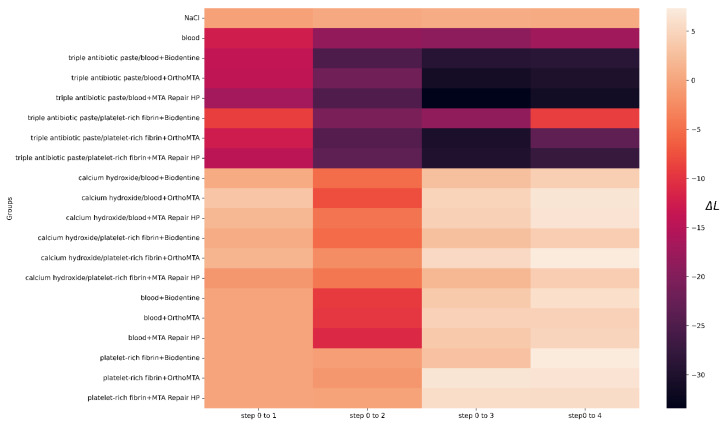
Values of ∆L for all groups and steps.

**Figure 6:**
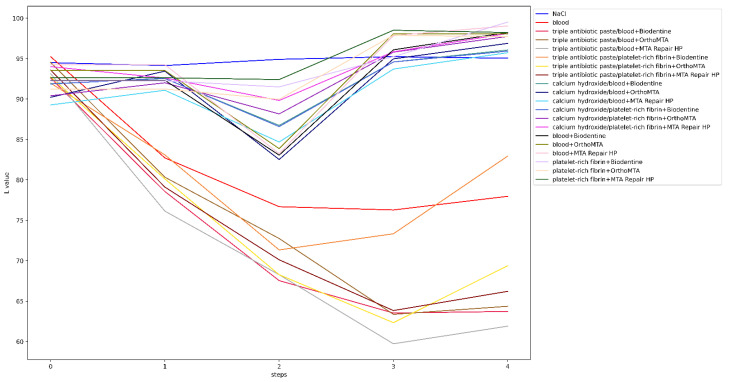
Values of L for all groups and steps.

**Figure 7:**
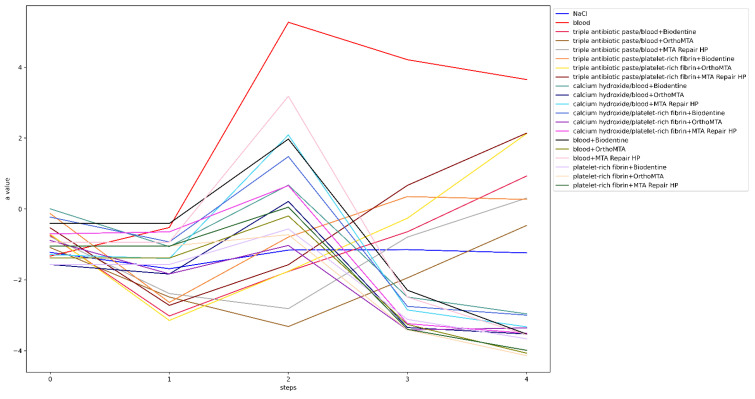
Values of a for all groups and steps.

**Figure 8:**
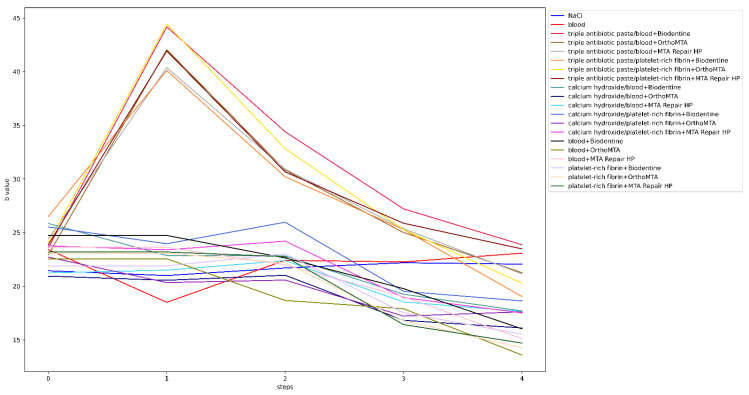
Values of b for all groups and steps.

**Figure 9:**
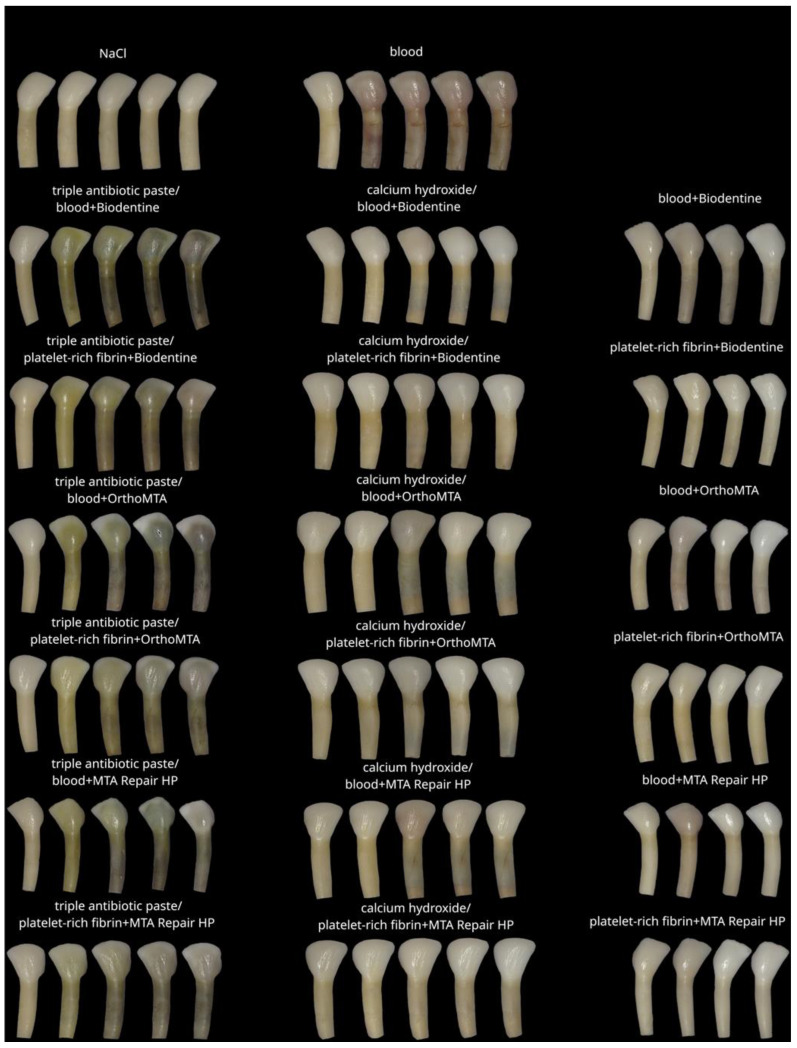
Representative images of the experimental groups. (**Left**), the first tooth after step 0; (**right**), the steps increase to step 4.

Before the intervention, one tooth from each of the following groups was rejected: groups 4, 13 and 20, due to a significant deviation from the initial colour. The lightness level distribution of the VITA SYSTEM 3D-MASTER across the steps is presented in Figure 3. In the qualitative analysis of group 1, no significant differences were observed between the steps (*p* = 1). The level of lightness in group 2 significantly differed between the initial and subsequent test steps. Means and standard deviations of the L, a, and b parameters for all groups for each step are presented in Table 4. The ∆E, ∆L, L, a, and b values for all groups and steps are shown in Figure 4, Figure 5, Figure 6, Figure 7 and Figure 8, respectively. Group 1 with NaCl did not present clinically perceptible discolouration at any stage of the study (ΔE < 3.3), unlike group 2 with blood (ΔE > 3.3). Photographs of all samples taken with the EyeSpecial C-II camera are shown in Figure 9.

Step 0 corresponds to the samples without any interference. In the qualitative analysis, most teeth were defined as lightness level 1 (Figure 3). The means and standard deviations for the values of L, a, and b are presented in Table 4. 

**Figure 10:**
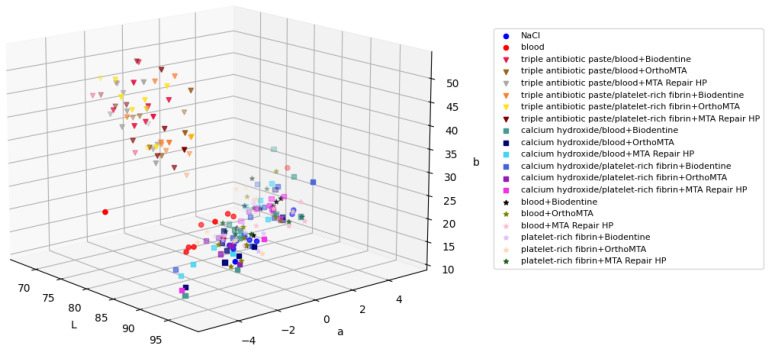
Three-dimensional scatter plot in the CIELab colour space (L, a, and b coordinates) of mean colour distribution of the groups after applying the disinfectant paste.

In step 1, visible tooth discolouration was observed in all groups in which the disinfectant pastes were applied. The mean value of ∆E for the groups with calcium hydroxide was 4.27, and for the groups with triple antibiotic paste, ∆E = 23.13. Significant differences in ∆E were found between the calcium hydroxide groups, triple antibiotic paste groups, and groups where the disinfectant pastes were omitted, but not within the groups themselves. Figure 10 shows the samples arranged in the first step in the three-dimensional CIELab colour space.

In step 2, after applying the scaffold and barrier material, the specimens of only one group, that is, group 20, showed no visible colour change (∆E = 2.62). Between steps 1 and 2, the highest ∆E value was reported for the groups in which triple antibiotic paste was used earlier. Despite the use of different scaffolds and barrier materials, no significant colour change was observed between the groups with triple antibiotic paste (*p* > 0.005). In step 2, the L value for groups with triple antibiotic paste decreased significantly compared with that in step 1. The ∆E value did not differ significantly in the triple antibiotic paste groups in which blood was applied as a scaffold, in comparison to the equivalent groups with calcium hydroxide and single-visit regenerative endodontic procedure groups. In the triple antibiotic paste groups in which platelet-rich fibrin was used, when compared to the matching groups without triple antibiotic paste, statistical significance was noted. When comparing the ∆E value in groups in which calcium hydroxide was applied previously and replaced with a scaffold and barrier material to the corresponding single-visit regenerative endodontic procedures groups, no significant statistical changes were noticed between steps 1 and 2. The lowest ∆E values in step 2 were achieved in the single-visit regenerative endodontic procedures groups with platelet-rich fibrin (group 20, ∆E = 2.62; group 18, ∆E = 3.94; group 19, ∆E = 4.40). Comparing the single-visit regenerative endodontic procedures groups between steps 1 and 2, a significant difference was noticed between the blood and platelet-rich fibrin groups. The BB, BO, and BM groups showed significantly more discolouration than the corresponding platelet-rich fibrin groups, but it was not significant compared to the positive control group (group 2).

**Figure 11:**
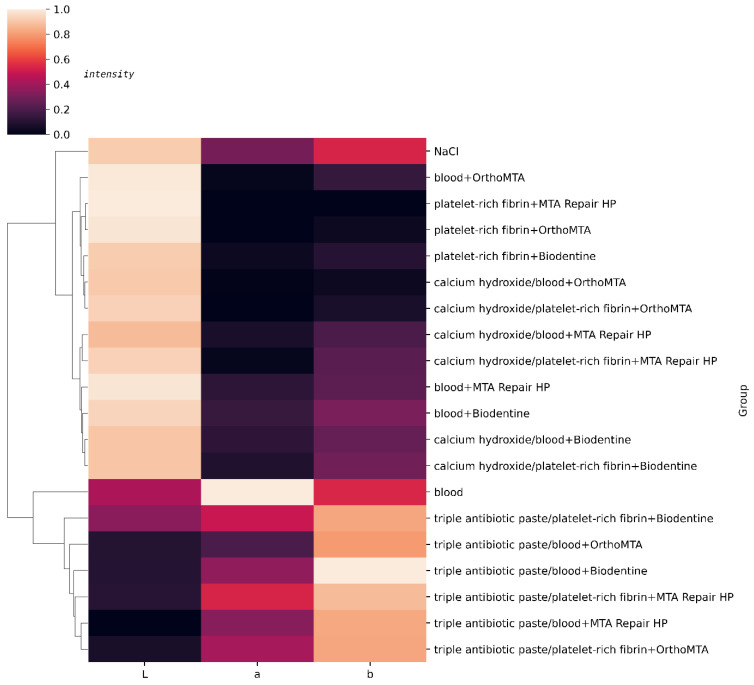
Hierarchical clustering dendrogram and heatmap for L, a, and b values after first bleaching procedure.

In steps 3 and 4, after the first and second bleaching procedure, each test group showed a visible change in the colour of the crown (∆E > 3.3) compared to the original shade. The L value decreased significantly in step 4 when compared to step 0 for the triple antibiotic paste groups. However, when comparing steps 4 to 0, we found that the L value in the groups with calcium hydroxide and single-visit regenerative endodontic procedures increased significantly. When analysing the ∆E value between steps 3 and 4, no changes in the colour of teeth were visually noticeable in the calcium hydroxide groups and group 20 (∆E < 3.3). Hierarchical clustering analysis was used to identify the colour groups formed from the L, a, and b values and to evaluate the colour change after the first bleaching procedure. Figure 11 shows the cluster dendrogram for L, a, and b values. Hierarchical clustering was used to group the samples according to the similar values of L, a and b in step 3. Group 1 and groups 3-8 formed one set. The second set included group 1 and groups 9–20. Each set was then divided into subsets to form the dendrogram in Figure 11.

## 4. Discussion

This study assessed the discolouration after regenerative endodontic procedures and the whitening effect after two-visit and single-visit regenerative endodontic procedures. The regenerative endodontic procedures were performed in accordance with the latest American Association of Endodontists guidelines of 2021, and the bleaching agent used in two sessions of whitening was in line with the General Dental Council’s Position Statement on Tooth Whitening guidelines for patients younger than 18 years of age. In this study, bleaching efficacy was analysed not only as tooth changes in brightness and colour, but also in terms of the possibility of returning to the original shade. Our study’s results were mostly in agreement with the null hypothesis of tooth discolouration after regenerative endodontic procedures and inconsistent with the whitening effects, which is not always achievable.

Human [31,32,34,49,50] and bovine [12,16,39,51] teeth are used in in vitro studies to evaluate tooth discolouration. Bovine incisors were used as the specimens in the present study. The diameter of the tubules in crown dentin and the number of dentinal tubules per mm^2^ in bovine teeth are similar to those in human teeth [52]; therefore, they can be used for studies in which the change in tooth colour is evaluated [53]. Additionally, the great advantage of using bovine teeth is the possibility of obtaining the appropriate number of teeth for extensive study. Changes in the colour of the teeth in the study were measured using a spectrophotometer. A similar technique has been used in other in vitro studies [31,32].

In the first step of this study, the colour of the teeth was evaluated 3 weeks after the application of disinfectant pastes. Specimens with triple antibiotic paste showed more significant crown discolouration than specimens with calcium hydroxide (*p* < 0.005). This finding is in agreement with the results obtained by other researchers that studied the discolouration potential of triple antibiotic paste [14,16,51]. It was noticed that despite rinsing the paste out of the root canal, the crown remained discoloured [54]; this may have been due to the fact that, regardless of the irrigation techniques used, 88% of the triple antibiotic paste remains in the root canal and is present at 350 µm in the dentin [55]. The authors noticed that discolouration appeared even 24 h after introducing the paste, which suggests that reducing the duration of antibiotic therapy does not prevent tooth crown discolouration [11,12,56]. The triple antibiotic paste samples had lower L and a values and higher b values, which made them darker, greener, and more yellow than the calcium hydroxide samples (Figure 10). Calcium hydroxide caused minor but perceptible changes in colour (∆E = 4.27). In an in vivo study, Chen et al. [13] observed discolouration in 2 out of 20 treated teeth using calcium hydroxide as a disinfectant paste in revascularisation procedures. Nagata et al. [57] used calcium hydroxide and chlorhexidine gel in 11 incisors, 3 of which were stained. However, in vitro studies using calcium hydroxide have shown that calcium hydroxide does not cause a colour change [14,16,51,58]. Before the disinfectant pastes were placed in the root canal, a dentin bonding agent was applied to the dentin in the pulp chamber to seal the dentinal tubules to create a surface that prevents any contamination [59] that may arise during the application of disinfectant paste and also prevents paste penetration into dentinal tubules. Despite the application of a dentin bonding agent, the tooth crowns became stained. This finding is in line with the results of studies by Kim et al. [11] and Shokouhinejad et al. [12], who noticed that applying a dentin bonding agent before placing triple antibiotic paste into the tooth does not completely eliminate discolouration, but reduces it. It is not fully known why dentin bonding agents do not prevent discolouration [60].

In the second step of this study, the colour of the teeth was evaluated 4 weeks after the scaffold (e.g., blood or platelet-rich fibrin) and barrier material (e.g., Biodentine, OrthoMTA or MTA Repair HP) applications. After this time, only group 20 showed no visible colour change (∆E = 2.62). ΔE values between steps 1 and 2 in the groups 3–8 were the highest and did not differ significantly between the groups. This finding is in line with the results presented by Shokouhinejad et al. [12], who observed that after replacing triple antibiotic paste with blood and barrier material, the teeth did not differ significantly between the groups. In all triple antibiotic paste groups, the L value decreased significantly and the teeth became darker in the second step, when compared to the first step. The lowest ∆E value in step 2 was observed in the groups 18–20. Out of these three groups, group 19 has the highest ∆E value. This may be due to the presence of bismuth oxide in OrthoMTA. Bismuth oxide is used as a radiopacifier and is associated with discolouration of dental tissues [61,62,63]. Oxidising agents, such as the amino acids present in dentin collagen or sodium hypochlorite, in contact with bismuth oxide destabilise it, which is associated with discolouration [64,65]. For the single-visit regenerative endodontic procedures groups, groups 15–17 showed more discolouration than the corresponding platelet-rich fibrin groups between step 1 and 2. Additionally, the results for groups 15–17 were not significantly different when compared to group 2. Based on these results, it can be concluded that blood has a greater staining potential than platelet-rich fibrin, regardless of the barrier material. Blood discolouration may be caused by the penetration of erythrocyte pigments into tooth tissues and the accumulation of decomposition products in the dentinal tubules [66]. The factor that increases ΔE may be the absorption of blood components by the freshly applied and unset barrier material [51]. Namazikhah et al. [67] noticed porosities in the microstructure of calcium silicate-based cement, which may take up blood components and cause discolouration of the material, and thus tooth discolouration [51]. The absence of erythrocytes in platelet-rich fibrin may be the cause of the decreased discolouration potential of platelet derivatives [39]. This result is in agreement with that reported by Shokouhinejad et al. [39], who found that the groups treated with blood showed more discolouration than the platelet-rich fibrin groups after 1 month. The difference between the studies was that Shokouhinejad et al. [39] used a double antibiotic paste that consisted of ciprofloxacin and metronidazole, but after 4 weeks, there was no visible change in tooth colour (ΔE ≤ 3.3). Statistical significance was noted in the triple antibiotic paste groups, in which platelet-rich fibrin was used as a scaffold, compared with the corresponding calcium hydroxide and single-visit regenerative endodontic procedures groups. This is due to the strong staining potential of triple antibiotic paste and the inability to completely rinse out the triple antibiotic paste, which means that it remains in the dentinal tubules [55] and could affect the colour changes in the tooth tissues. When comparing the triple antibiotic paste group, in which blood was applied as a scaffold, with the corresponding calcium hydroxide and single-visit regenerative endodontic procedures groups, the authors noticed that ∆E was higher in the triple antibiotic paste group, but it did not change significantly. The authors concluded that there may be a possible interfering effect of the triple antibiotic paste and blood staining potential due to a significant change in ∆E in the groups with platelet-rich fibrin when triple antibiotic paste was applied, but the difference was not significant in groups where blood was used. When comparing the colour change in the calcium hydroxide groups with the corresponding single-visit regenerative endodontic procedures groups, there was no statistical differences between steps 1 and 2; thus, it could be concluded that calcium hydroxide had no effect on further discolouration in step 2.

In the third and fourth steps of this study, the colour of the teeth was evaluated 1 week after the application of the bleaching material, which was applied twice. A 15% carbamide peroxide gel was selected for the study, which releases 5.25% hydrogen peroxide and urea [68] and is in line with the General Dental Council’s Position Statement on Tooth Whitening [25], assuming that regenerative techniques are most often conducted in children. Compared with other bleaching agents, carbamide peroxide is less cytotoxic to human dental pulp stem cells [69]. In the qualitative analysis, most of the teeth were defined as lightness level 0 in steps 3 and 4. Additionally, there were samples with lightness level 5 in step 1 (after disinfectant paste application), and their number increased in the following steps. Comparing steps 3 and 4 to step 0, in which most of the teeth were defined as lightness level 1, the authors concluded that after the first and second whitening procedures, the teeth did not return to their original colour. Most of the samples whitened too intensely, while some darkened. When analysing individual colour components, such as L, a, and b (Figure 6, Figure 7 and Figure 8), it was noticed that in the groups with triple antibiotic paste, the values of L and b decreased, whereas the value of a increased, which may mean that after the second whitening procedure, the teeth became darker, redder, and less yellow than the original colour. Based on a comparison of the brightness of the teeth colour before treatment and after the second whitening procedure, we concluded that in the groups with calcium hydroxide and single-visit regenerative endodontic procedures groups, the teeth became brighter, whereas in the triple antibiotic paste groups, the teeth became darker (Figure 5 and Figure 6). The results for the single-visit regenerative endodontic procedures groups were in line with those of Khedmat et al.’s study [32], which showed that the ΔL value increased after the second bleaching session in specimens in which blood and OrthoMTA were applied. The difference between the studies was the concentration of the bleaching agent, as Khedmat et al. [32] used 37% carbamide peroxide. The authors did not notice any visible (ΔE < 3.3) or statistically significant (*p* > 0.005) differences in the colour change in the tooth crown for any of the groups where calcium hydroxide was used, including group 20, between the first and second whitening procedure, which may mean that one whitening session is sufficient with this combination. In the remaining single-visit regenerative endodontic procedures groups, ΔE between steps 0 and 3 and steps 0 and 4 increased, suggesting an unfavourable whitening effect by increasing the colour change from the original colour.

Several in vitro studies have assessed tooth whitening after regenerative endodontic procedures. It is difficult to directly compare these studies with this study because different materials, specimen types, bleaching agents, and methods were used. Three authors used hydrogen peroxide [31,34,50], and one of the authors included the addition of sodium perborate [34]. Shokouhinejad et al. [50] analysed laser-assisted protocols. Only two studies analysed the effectiveness of carbamide peroxide in whitening discoloured teeth after regenerative endodontic procedures [30,32]. In an in vitro study using 37% carbamide peroxide gel, the authors found that reducing the size of the barrier material and placing glass ionomer cement as a cervical barrier in single-visit regenerative endodontic procedures may be helpful in reducing the number of whitening sessions [32]. Antov et al. [30] conducted an in vivo study in which teeth were whitened after regenerative endodontic procedures with 10% carbamide peroxide. In two cases, they used the technique of internal/external whitening using vacuum-formed bleaching trays. In the first case, the patient was satisfied with the whitening result; in the second case, there was minimal improvement in the shade, but the patient was no longer concerned about the discolouration. In the third case, the patient underwent an external bleaching technique, in which an improvement in the shade was noticed; however, the patient was not fully satisfied with the effect.

The extensive research group of 200 bovine teeth and the analysis of tooth discolouration after both single- and two-visit regenerative endodontic procedures, in which various scaffolds were used, including blood or platelet-rich fibrin and barrier materials such as Biodentine, OrthoMTA or MTA Repair HP, are the strengths of the study. Evaluation of teeth whitening for such an extensive research group with the use of a bleaching agent that is permitted for patients younger than 18 years of age is another advantage of this research.

This study has several limitations. In vitro conditions are not entirely compatible with in vivo conditions. Regenerative endodontic procedures are also performed under specific, controlled conditions, but they are not identical to those in the oral cavity; however, a sincere attempt was made to recreate a real-life situation. Additionally, in this study, the authors used triple antibiotic paste and calcium hydroxide as disinfectant pastes and three barrier materials in contact with blood and platelet-rich fibrin, which limits the assessment of discolouration and whitening to several combinations. Bleaching was carried out using the internal technique with the use of one bleaching material at a specific concentration. It is recommended that further studies carry out a procedure using other bleaching agents with an acceptable concentration and with a different whitening technique. Randomised clinical trials and long-term studies are needed to establish accurate post-regenerative endodontic procedure tooth-whitening guidelines.

## 5. Conclusions

Discolouration of dental tissues after regenerative endodontic procedures is almost inevitable. It is more advisable to use platelet-rich fibrin as a scaffold. It is worth considering whitening discoloured teeth after single- and two-visit regenerative endodontic procedures with calcium hydroxide because it is achievable, but it does not guarantee a return to the original colour. It is not recommended to whiten teeth that have undergone treatment with triple antibiotic paste, as the whitening effect will be opposite to the desired effect. Further studies are required to create precise guidelines to define the appropriate whitening technique, material, and duration of discolouration after regenerative endodontic procedures. 

## Figures and Tables

**Table 1:** Compositions of barrier materials.

Material	Manufacturer	Compounds	Radiopacifier	Mixing
Biodentine	Septodont, Saint-Maur-des-Fossés Cedex, France	Powder: tricalcium silicate, dicalcium silicate, calcium carbonate and oxide filler, iron oxide shade, and zirconium oxide Liquid: calcium chloride as accelerator, hydrosoluble polymer water-reducing agent, and water	Zirconium oxide	0.7 g capsule of powder + 5 drops of liquid (triturator): 4000–4200 rpm; 30 s
OrthoMTA	BioMTA, Seoul, Korea	Powder: calcium carbonate, silicon dioxide, aluminium oxide, and dibismuth trioxide	Dibismuth trioxide	0.2 g pouches of powder + 2 drops of water mixed manually
MTA Repair HP	Angelus, Londrina, PR, Brazil	Powder: tricalcium silicate, dicalcium silicate, tricalcium aluminate, calcium oxide, and calcium tungstate Liquid: water and plasticizer	Calcium tungstate	0.085 g capsules of powder + 2 drops of liquid mixed manually

**Table 2:** Measurement time points for a specific step.

Step	Measurement Time Points
0	Before treatment
1	3 weeks after placement of disinfectant pastes
2	4 weeks after application of scaffold and coronal barrier
3	1 week after first bleaching procedure
4	1 week after second bleaching procedure

**Table 3:** Shades of the Toothguide 3D Master grouped into five groups of lightness levels.

Lightness Level	Shade
0	0M1, 0M2, and 0M3
1	1M1 and 1M2
2	2M1, 2L1.5, 2R1.5, 2M2, 2L2.5, 2R2.5, and 2M3
3	3M1, 3L1.5, 3R1.5, 3M2, 3L2.5, 3R2.5, and 3M3
4	4M1, 4L1.5, 4R1.5, 4M2, 4L2.5, 4R2.5, and 4M3
5	5M1, 5M2, and 5M3

**Table 4:** Means and standard deviations of L, a, and b values for all groups for each specified step.

	Step 0	Step 1	Step 2	Step 3	Step 4
L (mean ± SD)	92.617 ± 3.220	88.211 ± 7.083	81.544 ± 9.870	85.480 ± 15.439	87.598 ± 14.999
a (mean ± SD)	−0.879 ± 1.170	−1.632 ± 1.499	0.022 ± 2.585	−1.755 ± 2.361	−1.724 ± 2.866
b (mean ± SD)	23.484 ± 3.640	28.252 ± 10.248	25.143 ± 5.499	20.856 ± 4.775	18.452 ± 4.937

SD, standard deviation.

## Data Availability

Data available upon request due to restrictions, e.g., privacy or ethical. The data presented in this study are available upon request from the corresponding author. The data are not publicly available due to technical and logistic issues.
